# Screening and diagnosis of COPD and asthma based on government guidelines empowering peripheral health workers in Pune district Maharashtra, India: A study protocol

**DOI:** 10.1371/journal.pone.0308210

**Published:** 2024-09-05

**Authors:** Jayashree Sachin Gothankar, Medha Deepak Bargaje, Sanjivani Vishwanath Patil, Prakash Prabhakarrao Doke

**Affiliations:** 1 Department of Community Medicine, Bharati Vidyapeeth Deemed University Medical College, Pune, Maharashtra, India; 2 Central Research and Publication Unit, Bharati Vidyapeeth Deemed University Medical College, Pune, Maharashtra, India; 3 Department of Pulmonary Medicine, Bharati Vidyapeeth Deemed University Medical College, Pune, Maharashtra, India; Public Library of Science, UNITED KINGDOM OF GREAT BRITAIN AND NORTHERN IRELAND

## Abstract

COPD is the second leading cause of death in India. The guidelines for early detection of COPD were released by the Government of India in 2019. However, due to the COVID-19 pandemic, its implementation could not be optimal. Diagnosis of COPD is based on the presence of respiratory symptoms, the presence of exposure to risk factors, and the presence of poorly reversible airflow obstruction as assessed using a spirometer. Spirometers are currently available only at a few district hospitals. The existing guidelines expect the patient to visit the Rural hospital/ Community Health Centre, which does not have a spirometer or a pulmonary medicine specialist. Also, it is not feasible or accessible for patients to visit the district hospital to get diagnosed. The current study will be implemented to determine the prevalence, annual incidence of COPD and asthma, quality of life, and nutritional status of COPD and asthma patients. The novelty of this implementation research, which will be conducted in collaboration *with Zilla Parishad* (i.e., Government), Pune district, is the empowerment of an Accredited Social Health Activist (ASHA), a peripheral health worker to screen all individuals using a peak flow meter and confirmation of the diagnosis at health and wellness center (HWC). An accredited Social Health Activist (ASHA) will take relevant history to suspect COPD and asthma in 30+-year-old adults, and she will refer the suspected cases to the Community Health Officer (CHO) at the Health and Wellness Center. The CHO/ Medical officer of PHC will initiate the appropriate treatment after confirming the diagnosis using a portable spirometer. The difficult-to-diagnose patients with comorbidity and acute exacerbations will be referred to the nearest higher center, i.e., Primary Health Centre (PHC) or Community Health Centre (CHC), where a primary care physician is available. The ASHA workers will provide two follow-ups to these patients in a year, depending on the severity, to ensure compliance with the treatment. Thus, early diagnosis and appropriate treatment of COPD and asthma at the community level may help to reduce the episodes of acute exacerbations.

## Introduction

Chronic respiratory diseases (CRDs) are diseases of the airways and other structures of the lung. The commonest are chronic obstructive pulmonary disease (COPD), asthma, and occupational lung diseases. According to the Global Burden of Disease Report (2019), in India, there are an estimated 37.8 million cases of COPD, contributing to 17.8% of the global burden [1]. COPD is the second leading cause of death and DALYs in India. Although India contributes to 17.8% of the global burden of COPD, it contributes to a disproportionate 27.3% of the global deaths, indicating a lack of standard treatment [2]. Similarly, India contributes to 13% of the global asthma burden, and it contributes to a disproportionate 43% of the global asthma deaths, indicating that asthma remains poorly managed in India [1]. This has a significant long-term implication in terms of increasing burden to the patient, family, community, health care system, and economy [3]. The diagnosis of COPD requires a broader approach, which includes assessment based on symptoms and risk factors, namely smoking, domestic and occupational exposure to smoke, and Spirometry [4]. Even though Spirometry is the gold standard for diagnosing COPD, it still needs to be utilized at the primary care level [5]. Patient access to diagnostic and management facilities, drug therapies, and non-pharmacological interventions like pulmonary rehabilitation needs to be improved. If the conditions are not adequately managed, the frequency of exacerbation of COPD is high, and the severity of exacerbations can be assessed by pulse oximetry [6].

The Government of India formulated guidelines in 2019 for the prevention, early detection, and management of COPD and asthma diseases for use in the public health system [2]. However, the implementation was hindered due to the COVID-19 pandemic. These guidelines define the roles and responsibilities of ASHA Workers in the prevention and early detection of COPD and Asthma; however, her role is minimal [2]. An earlier work done in this area has shown that trained ASHA can suspect COPD or asthma with reasonable accuracy using a peak flow meter [7]. Timely and accurate diagnosis of COPD will have benefits both in short-term and long-term outcomes [8]. Early diagnosis and treatment of such patients will improve the quality of life of the patients and reduce Disability Adjusted Life Years (DALY), mortality, and health care expenses. The Government plans to provide a spirometer at the RH/ CHC level. However, considering the field experience, it will be needed at the most peripheral institution, i.e., HWC, which will be available in the proposed study.

## Material and methods

The study aims to estimate the baseline prevalence and annual incidence of COPD and Asthma among adults aged 30 years and above from rural areas and to assess the severity of disease, quality of life, and nutritional status of COPD patients at baseline and every two years during the project’s implementation.

### Study design

Implementation study.

### Study setting

The study will be implemented in the villages under the *Male* PHC area of Mulshi block of Maharashtra, India.

### Study duration

Three years.

### Sample size

The study will include all male and female individuals aged 30 years and above who are permanent residents of the villages under *the Male* Primary Health Centre (PHC) and give consent. There will be about 14,000 estimated participants. The *Male* PHC has eight Health and wellness centers (HWC), including one each in *Male* PHC and Paud Rural Hospital. Community Health Officers (CHO) oversee HWC. It is expected that around 1,400 participants will be diagnosed with COPD/asthma through this study. Most patients will be old and otherwise unlikely to go to the district hospital for treatment. With adequate capacity building, all the mild-moderate cases from the above-estimated number can be diagnosed and adequately treated at the HWC level. In contrast, the severe cases can be referred to PHC/ CHC. Table 1 gives an expected number of participants per ASHA and *Male* PHC.

**Table 1:** Total population and expected number of participants per ASHA and *Male* PHC in Mulshi block at various levels of study.

Number	Per ASHA	Per *Male* PHC
Total population catered	1000	35000
Participants (≥30 years of age-40% of total) to be screened by ASHA through house-to-house visits	400	14000
Participants that will be suspected and referred (approx.20% of B) to HWC	80	2800
Participants who will be confirmed with COPD/ asthma based on Spirometry and counseled/ trained in inhalation techniques	40	1400

#### Inclusion criteria

All permanent residents (residing more than six months or who intend to stay for more than six months in the village) of the villages aged 30 years or above and who consent to participate will be included.

#### Exclusion criteria

Terminally ill patients.

### Variables to be measured

Essential sociodemographic variables, namely age, sex, education, occupation, socioeconomic status; type of fuel used for cooking purposes, smoking status, occupational exposure to smoke, history of chronic cough, assessment of breathlessness if present; quality of life, Body mass index, Peak expiratory flow rate (PEFR) using peak flow meter, whether known case of COPD or asthma. COPD will be defined as symptoms of dyspnoea, cough, sputum production, and exacerbations due to abnormalities of airways and alveoli leading to airflow obstruction and post-bronchodilator FEV1/ FVC < 0.7 as per Spirometry [9]. Asthma diagnostic criteria are wheezing, shortness of breath, chest tightness, and/ or cough with confirmed variable expiratory airflow limitation, i.e., positive bronchodilator (BD) responsiveness (reversibility) test with Spirometry (or PEF1)seen as an increase of FEV_1_ or FVC ≥12% and ≥200 mL predicted compared to pre-BD reading. These changes are measured 10–15 minutes after 200–400 mcg of salbutamol Short-acting beta-agonists (SABA). The SABA is to be withheld for 4–6 hours, and Short-acting muscarinic-antagonist (SAMA) for 12 hours before Spirometry) [9]. The COPD Assessment Test (CAT) scale will be used to assess the effect of COPD on patients’ lives and will be applied only to known cases of COPD. It is a questionnaire with eight items to assess the health status of a COPD patient. The maximum score is 40, and the minimum is 0 [8]. The mMRC scale for assessment of dyspnoea: Dyspnoea level will be measured using modified Medical Research Council (mMRC) scale. This scale is easy to use and has a prognostic value.

Table 2 gives the details of the workforce in the study.

**Table 2:** Human resources in the project.

Sr. No.	Category	Number
I	**Government functionaries**	
	ASHA workers	35
	ASHA Supervisor	02
	Auxiliary Nurse Midwife (ANM)	04
	Community Health Officer	06
	Medical Officer Primary Health Centre	01
	Medical Officer Rural Hospital	03
II	**Project staff (Full time)**	
	Senior Project Scientist	01
	spirometry technician cum Senior supervisor	01
	Senior supervisor	01
	General support staff	01

Other stakeholders: District Health Officer, Taluka Health Officer of Mulshi Block

**The instruments to be used in the study** are a validated and pretested proforma, a peak flow meter, a height measuring machine, a weighing machine, a pulse oximeter, a Peak flow meter, a CAT scale, an mMRC scale, and a portable spirometer.

### Methodology

The District Health Officer (DHO)and Mulshi Taluka Health Officer (THO) are both sensitized toward the objective and activities of the project. Their cooperation is sought for the successful implementation of the project. A meeting with the PHC medical officer was held in the project’s planning phase. Similarly, a meeting with Community Health officers, ANM, ASHA supervisors, and ASHA was conducted to explain their roles and responsibilities in the project. A letter to *Sarpanch* (Head) of all the villages is given, and community leaders are contacted, and their cooperation is sought. The project’s human resources are required at various levels and are interviewed and recruited. The Primary Health Centre (PHC) Medical Officer (MO) and Community Health Officer (CHO) are trained for two days on topics such as information about the project and their roles and responsibilities in it, risk factors for COPD and asthma, and signs and symptoms of COPD and asthma. Training also included hands-on training regarding using a spirometer and identifying difficult/ severe cases for timely referral to higher centers, treatment modalities, and counseling of patients. The 35 ASHA workers are trained for the baseline survey and enrolment, including using and interpreting a peak flow meter, pulse oximeter, Height and weight measurement, and referring suspected cases. They will be trained to follow up with diagnosed patients and receive health education on risk reduction.

Implementation of the project at the Community Level: Household visits- A baseline survey and screening by ASHA workers of all ≥ 30-year-old permanent village residents will be done. She will collect sociodemographic information, enquire about smoking status and occupational exposure to smoke, assessment of chronic cough and breathlessness if present. She will determine the PEFR of all the eligible participants using a peak flow meter. She will measure the oxygen saturation of known cases of COPD/asthma using a pulse oximeter and collect information about the history of exacerbations in the last year. In addition, ASHA will measure Height and weight in a standard manner to calculate the Body Mass Index. She will also assess the quality of life using the EQ-5D questionnaire. This screening will be done in the first quarter of all three years of the project. Data collection will be done using an application on ASHA’s mobile phones, and she also perform hard copy data collection after obtaining consent for participation and publication of the anonymized data from the eligible participants. Participants who are smokers or have occupational exposure to smoke/ dust; have a cough of more than eight weeks duration; have Peak expiratory flow rate (PEFR) reading less than predicted for the given age, sex, and height standards will be labeled as "suspected cases" and will be referred to the nearest HWC for further assessment. Known cases of COPD/ Asthma with low oxygen saturation level (<95%) or participants having symptoms suggestive of Tuberculosis or other diseases will be advised referral by the ASHA. She will give the referral note mentioning the participant’s CAT score, Height, and weight. At the Health and Wellness Centre level- The suspected cases referred by ASHA to the nearest HWC will undergo Spirometry to confirm diagnosis and grade the disease based on Global Obstructive Lung Disease (GOLD-2024) criteria (permission obtained) and mMRC score. Contraindications for Spirometry are Haemoptysis of unknown origin, pneumothorax, recent myocardial infarction or pulmonary embolus, recent eye surgery (e.g., cataract), Presence of an acute illness or symptom that might interfere with test performance (e.g., nausea, vomiting), active TB, recent thoracic or abdominal surgery. Similarly, Tuberculosis in the suspected patients will be ruled out. Difficult-to-diagnose patients will be referred to the nearest PHC/RH/District hospital. CHO will advise treatment for individuals diagnosed with COPD and/ or asthma per the existing government guidelines. Health education on inhaler techniques and risk reduction (quitting tobacco and using safe fuel for cooking whenever necessary, balanced diet, and regular exercise) will be provided to the patients diagnosed with COPD and / or Asthma at HWC. Patients will be advised to go to a higher centre in case of deterioration or no relief.

Follow-up of suspected and diagnosed cases- At least two follow-up visits to patients diagnosed with COPD and/or asthma within one year will be provided by the ASHA with intervals of one to three months based on the severity of the disease. During her visit, the ASHA will look for patient compliance with the inhaled drugs and fill out a checklist that will be provided; she will also counsel the patient. She will assess breathlessness using the mMRC scale, monitor oxygen saturation using a pulse oximeter during these follow-up visits, and refer patients with exacerbations to the nearest HWC/PHC. At PHC/ CHC/ District Hospital- The medical officer of RH/DH will recommend pharmacotherapy based on the diagnosis and severity of the disease, as per the guidelines. Confirmation of diagnosis in severe/ uncertain cases. In the proposed study, the healthcare delivery level for COPD and asthma will be modified, i.e., Spirometry will be done at the HWC level.

### Quality checks

Data collection by ASHA will be supervised by the ASHA supervisor through her visits to each ASHA. A checklist will be shared with the ASHA supervisor, and she will do random cross-checking of the data. The spirometry technician and senior supervisor will check the tests, especially the peak flow meter, and ensure the referrals. The senior supervisor will monitor the cases at the HWC levels, too. Critical areas in the data collection tool will be identified and monitored. The entry into the application will be monitored from the headquarters level. The data of 5% of the individuals will be verified at all levels.

### Data management plans

Data collection forms for the study have been developed. These are the ASHA baseline tool, Community Health Officer (CHO) tool, ASHA follow-up tool, referral tool, and Training tool. The language of these tools is *Marathi*. All tools are validated and pretested. Weekly data download will be done. A unique code will be assigned to each individual. All data will be in the custody of the study’s Principal Investigator.

### Primary outcome measures

The baseline prevalence and annual incidence of confirmed cases of COPD and asthma will be measured at the end of the first year of the study

### Secondary outcomes

The percentage of patients with acute exacerbations of COPD and asthma, the compliance/ medication adherence rate amongst COPD and asthma patients, and the quality of life of the COPD and asthma patients at baseline and for two years using the EQ—5D questionnaire.

### Statistical analysis planned

All statistical Analysis will be done using SPSS software with version 29. Descriptive statistics will show the results of the quantitative variables, and the results of the qualitative variables will be shown by frequency and percentages. Graphs will be added wherever necessary. The chi-square test will test the association between demographic variables, risk factors like smoking, occupational exposure, family history, use of biomass fuel, and COPD and asthma. Bivariate and multivariate analysis will be done to find the prevalence ratio and adjusted the prevalence ratio using logistic regression analysis. A 5% significance level will be used throughout the results. Paired t-test/Wilcoxon sign rank test will be used to test significant mean/median difference between Quality of life at baseline and yearly follow-up.

### Ethical issues

The final study protocol, including the final version of the other essential documents, is approved by the Institutional Ethics Committee (DHR Reg. No: EC/New/INST/2022/MH/0150) approval number BVDUMC/IEC/04 dated 28/09/2023.

Written informed consent for participation and permission to publish the anonymized data will be obtained.

**Fig 1:**
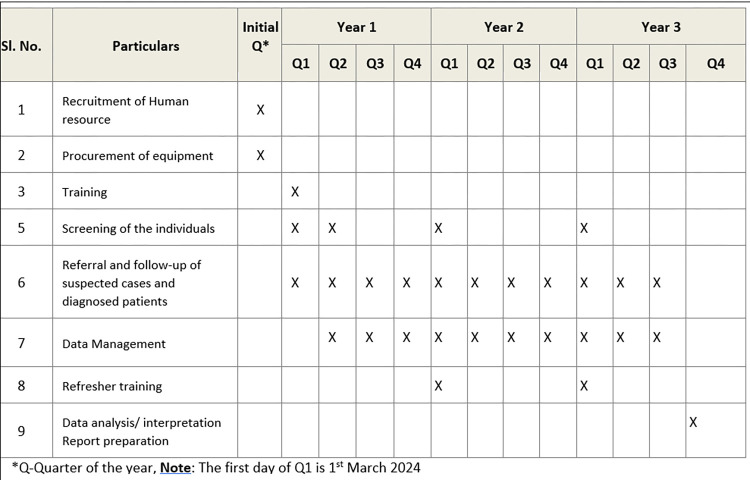
Status and timeline of the study.

**Fig 2:**
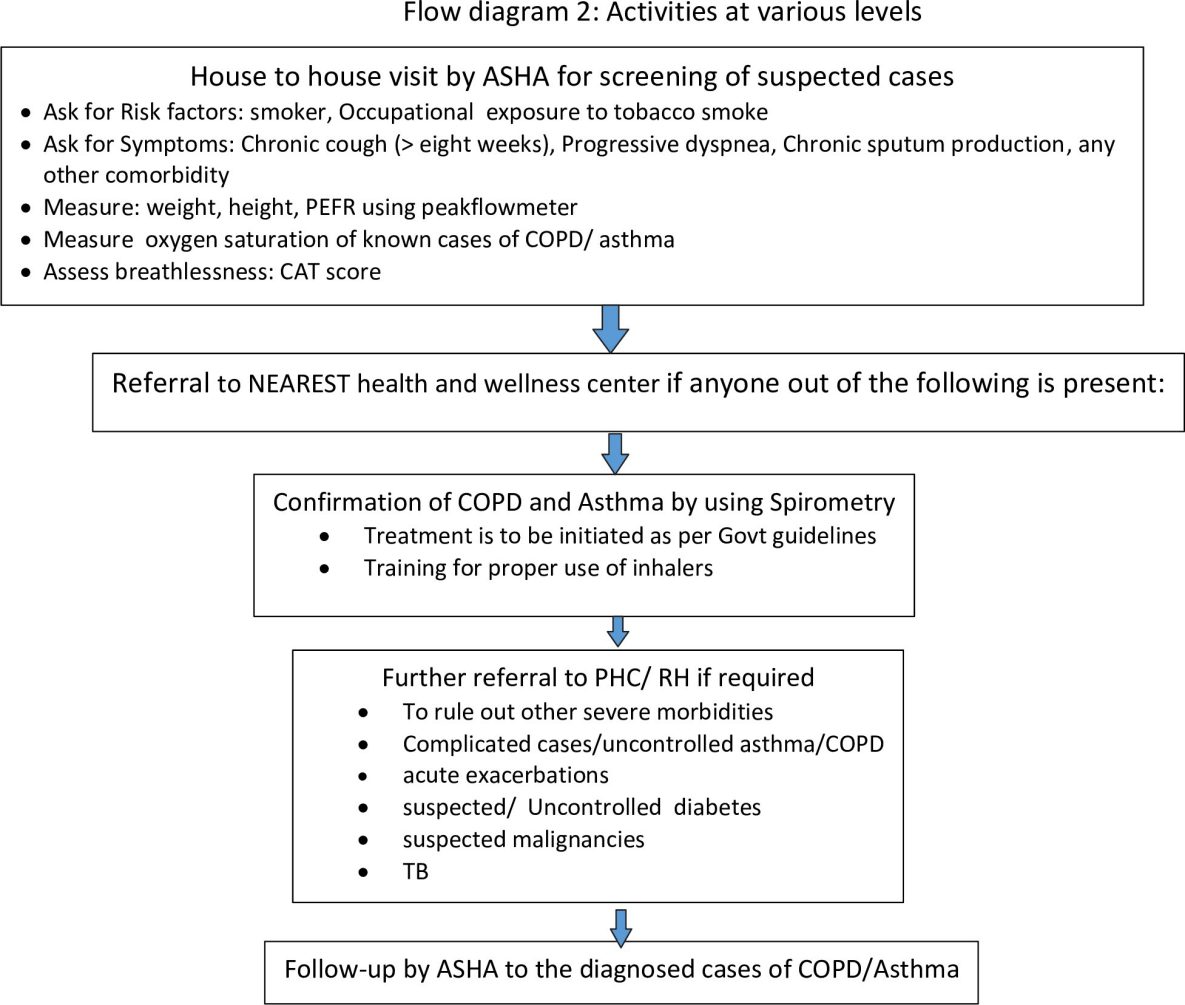
Depicts the activities at various levels.

Currently, the study is in the Q1 of year one phase (Fig 1), i.e., the recruitment of participants or data collection for the study has not been completed. The activities at various levels have been initiated (Fig 2).

## Discussion

This study will be implemented by a private university medical college with the help of government voluntary health workers (ASHA) who will screen for COPD/asthma through house-to-house visits. The government health officers (MO PHC and CHO) will confirm the disease condition. The study will only diagnose COPD and asthma. The routine healthcare system will take care of the treatment part.

### Dissemination plans

COPD is a national priority disease. The study’s results will be shared with the Government and may be scaled up to other districts/ states in India.

## Abbreviations

COPD: Chronic Obstructive Pulmonary Disease
CRD: Chronic Respiratory Disease
CHO: Community Health Officer
PHC: Primary Health Centre
HWC: Health and Wellness Center
PEFR: Peak Expiratory Flow Rate
ANM: Auxiliary Nurse Midwife
ASHA: Accredited Social Health Activist
RH: Rural Hospital
THO: Taluka Health Officer
DHO: District Health Officer
mMRC: Modified Medical Research Council
CAT: COPD Assessment Test
BD: Bronchodilator
SABA: Short-acting beta-agonists
SAMA: Short-acting muscarinic-antagonist
